# Pet Owner Perception of Ferret Boredom and Consequences for Housing, Husbandry, and Environmental Enrichment

**DOI:** 10.3390/ani12233262

**Published:** 2022-11-23

**Authors:** Alice M. M. Dancer, María Díez-León, Jennifer K. Bizley, Charlotte C. Burn

**Affiliations:** 1 Department of Pathobiology and Population Sciences, The Royal Veterinary College, Hawkshead Lane, North Mymms, Hertfordshire, Hatfield AL9 7TA, UK; 2 Ear Institute, University College London, 332 Gray’s Inn Road, London WC1X 8EE, UK

**Keywords:** animal welfare, affective state, boredom indicators, companion animals, positive reinforcement training

## Abstract

**Simple Summary:**

Boredom may be an overlooked animal welfare problem because of monotonous or predictable routines and confined living conditions that often typify captivity. We investigated whether pet ferret owners believe ferrets are able to experience boredom and which behaviours they use to recognise ferret boredom using an online questionnaire. We also explored whether owners’ beliefs of ferret boredom were linked to environmental enrichments (e.g., toys and shelters) or housing they provided or the style of training they used for their ferrets. Of the 621 responses, most (93%) owners believed that ferrets could experience boredom. Owners who doubted that ferrets could feel bored gave their ferrets significantly fewer types of environmental enrichment than other owners did. The analysis of behaviours that owners linked with boredom showed that ferrets ‘scratching at enclosure walls’ and ‘sleeping more than normal’ were key behaviours that owners use to distinguish ferret boredom from other emotions. This fits with the idea that boredom causes both active seeking behaviour and excessively inactive behaviour. Owners suggested housing with other ferrets, human interaction, and exploration as most important for preventing boredom. These results suggest that pet ferrets are at risk of poorer welfare if their owners doubt that ferrets can experience boredom.

**Abstract:**

Boredom is a potential chronic but overlooked animal welfare problem. Caused by monotony, sub-optimal stimulation, and restrictive housing, boredom can therefore affect companion animals, particularly those traditionally caged, such as ferrets. We surveyed owners’ (*n* = 621) perceptions of ferrets’ capacity to experience boredom, behaviours they associate with it, and whether their perception of their ferrets’ capacity for boredom influenced training techniques, housing, and environmental enrichment (EE). Most (93.0%) owners believed that ferrets could experience boredom, but owners who doubted that ferrets experience boredom (7.0%) provided slightly but significantly fewer EE types to their ferrets. Heat map and classification tree analysis showed that owners identified scratching at enclosure walls (*n* = 420) and excessive sleeping (*n* = 312) as distinctive behavioural indicators of ferret boredom. Repetitive pacing (*n* = 381), yawning (*n* = 191), and resting with eyes open (*n* = 171) were also suggested to indicate ferret boredom, but these overlapped with other states. Finally, ferret owners suggested social housing, tactile interaction with humans, and exploration as most important for preventing boredom. These results suggest that pet ferrets are at risk of reduced welfare from owners who doubt they can experience boredom, highlighting an opportunity to improve welfare through information dissemination. We recommend further investigation into ferret boredom capacity, behavioural indicators, and mitigation strategies.

## 1. Introduction

Animal-boredom research is in its relative infancy, and knowledge of how pet owners perceive and understand animals’ capacities to experience boredom is even smaller still. Predictable, monotonous, and restrictive conditions can result in boredom, a negatively valanced affective state (reviewed in [1,2]). Boredom can be defined as an “aversive experience of wanting, but being unable, to engage in satisfying activity” [3], and it includes a motivation for almost any stimulation or behavioural possibilities not currently available. For captive non-human animals, daily life can often be highly monotonous and unstimulating. For these animals, boredom is likely to be a prevalent, serious, and chronically over-looked welfare issue [1,4]. Despite this, animal boredom, including its causes, effect on individuals, identification, measures, and means of mitigation or prevention, have been little studied, with few research articles explicitly exploring boredom-like behaviour experimentally in animals to date [5,6,7,8]. This work has thus far focused on mink and ferrets, both mustelids, and the current study continued to build upon this.

### 1.1. Human Beliefs about Animal Boredom

Some authors suggest that humans may be the only species that can experience boredom (e.g., [9,10]). However, there is also some evidence that leads people to believe that other animals are capable of boredom, for example, pet horses [11], pet dogs, pet cats, farmed pigs, and—to a lesser extent—farmed cows [12]. Establishing pet owners’ beliefs of animal boredom could be a crucial tool in improving pet welfare, because people’s perceptions of animals’ mental abilities and capacity to experience emotion can directly affect animal welfare. Indeed, rabbit pet owners who had increased perceptions of rabbit emotion, intelligence, and pain were more likely to house and manage rabbits in higher welfare conditions through the provision of a rabbit partner, varied environmental enrichment (additions or improvements to an animal’s environment to enable greater behavioural diversity and a heightened wellbeing [13,14,15,16]), and a suitable diet and housing type [17]. Additionally, cat owners with a greater knowledge of cats’ emotional and intellectual abilities were less likely to use positive punishment (applying an aversive stimulus to an animal after the performance of an undesired behaviour) to reduce future occurrences of the behaviour (as defined in [18]) when training their cats than owners with poorer knowledge [19]. The use of aversive training methods, such as punishment, can be detrimental to animals both physically and mentally [20]. McMahon and Wigham [17] suggested that improving pet owners’ perceptions of the intelligence and emotional capabilities of rabbits could be a practical way of improving rabbit housing and management, and this may be the same for other species too. If pet ferret owners’ perceptions of ferret boredom were found to be associated with their decisions around ferret housing, husbandry, and management, then, similar to the recommendations made by McMahon and Wigham [17] for rabbits, raising pet ferret owners’ awareness of ferrets’ emotional abilities could also be a useful tool in promoting higher welfare for pet ferrets.

### 1.2. Pet Ferret Housing, Husbandry, and Behaviour in Relation to Levels of Stimulation

Ferrets (*Mustela furo*) are a domesticated mustelid species and are a popular pet, with an estimated 100,000 pet ferrets in the UK [21] and an estimated 501,000 in the USA [22]. Ferrets are considered to be a gregarious, explorative, and neophilic species [23], traits which may predict a propensity to experience boredom (reviewed in [1,24]). However, pet ferrets are still often housed and managed in ways that may put them at risk of boredom, as shown by data collected in a survey related to the current study [13], where 62.5% (*n* = 621) of pet owners kept ferrets in cages, rather than larger enclosures. Restrictive housing, such as cages, could pose a risk to ferrets of suffering from boredom when suitable environmental enrichment is not regularly provided, such as little or no time allowed exploring outside of the home cage or a lack of provision of behaviourally relevant stimulation, such as digging substrates or tunnels. Indeed, there was a large disparity in the amount of environmental enrichment provided by pet ferret owners, with some reporting providing as many as 38 types of enrichment and others as few as two types of enrichment, with a median provision of 16 types of environmental enrichment [13]. 

Providing low numbers of environmental enrichment types, alongside limited exploration time outside of housing and a restrictive housing size, can result in ferrets showing abnormal behaviours and increased biting, as indicated in previous surveys [25,26]. This is consistent with poor welfare in ferrets, with boredom being one potential outcome. Yet, boredom can be mitigated against; by providing just an hour’s exploration time, with access to environmental enrichment outside of their cage, behavioural signs of boredom in laboratory ferrets were reduced even 24 h later [6]. In a related species, the American mink (*Neovison vison*), those housed with environmental enrichment (running water and various manipulable and structural items) and a larger cage showed less interest in the stimuli presented in sensation-seeking tests than those housed in fur farm standard non-enriched cages, indicating that the increased space and environmental enrichment reduced boredom-like behaviour [7,8]. 

The environmental enrichments presented to ferrets could be a valuable means of mitigation against boredom. Novelty is important for mitigating boredom in both human and non-human animals [1,27]. Therefore, effective boredom mitigation strategies could include offering voluntary opportunities to explore novel locations and objects, which provide stimulation and bring unpredictability into the ferrets’ captive routine. Additionally, providing ferrets with cognitive stimulation, such as working to access food through devices such as puzzle feeders or providing scent trails [13], could help mitigate boredom through providing a meaningful challenge—working to access reward—and engaging the ferrets’ cognitive processes [28,29]. To evaluate whether interventions to mitigate animal boredom are effective, it is important to monitor the effects on that animals’ welfare using relevant behavioural or other indicators.

### 1.3. Indicators of Boredom in Animals

Understanding how specific emotions are manifested in non-human animals can be problematic without the self-reporting measures available in the study of human emotion because how emotions are experienced are private to the individual (e.g., [30,31]). The study of animal boredom has additional challenges, with animals in boredom-like states exhibiting both high arousal (e.g., restlessness) and low arousal (e.g., lethargy) behaviours (see [1,32]). Measuring boredom is further complicated by potential behavioural indicators also occurring with other affective states, such as ‘inactivity’ with depression, apathy, or relaxation [33]. However, in combination, indicators of boredom present a distinctive profile of both arousal-seeking and low arousal behaviours. Boredom indicators might therefore comprise a combination of (a) arousal-seeking behaviours (e.g.; restlessness, distractibility, unprovoked aggression, or escape behaviour) and (b) low arousal behaviour (e.g., drowsiness, lying awake inactive, and yawning), because the associated arousal state is both aversive and sub-optimal [1]. 

Empirical identification of the behaviours that reliably occur when an animal is experiencing boredom is crucial for both adding to our understanding of animal boredom and for improving animal welfare. Therefore, pet owners who have a close relationship with and who regularly observe their ferrets may provide a useful hypothesis-generating source of information for the indicators of boredom-like behaviour, which can then be compared against those proposed from a theoretical basis in the literature [1]. Agreement between pet ferret owners and the literature would provide face validity to boredom-like behavioural indicators, while disagreement could signify a need for further investigation into which indicators of boredom in ferrets are meaningful before use in future experimental boredom studies.

### 1.4. Aims and Hypotheses

Consequently, the aims for this study were to use an online questionnaire to describe to what extent ferret caretakers believe that ferrets experience boredom and to investigate how pet ferret owners’ beliefs about ferret boredom are associated with their provision of environmental enrichment, housing type, and training style. As previously mentioned, studies have identified that pet owners who believe their pets’ to have a greater capacity for emotions provide more appropriate housing and more environmental enrichment types, and they use less aversive training styles than pet owners with a more limited perception of their pets’ emotional abilities. Therefore, we developed the following hypothesis: if the pet owner provision of appropriate husbandry is at least partly motivated by their belief in their pet’s emotional capacity, then pet ferret owners who express doubt about their pets’ capability of experiencing boredom will report keeping ferrets in more basic housing, providing less time out of enclosure, providing fewer environmental enrichment items, and training using more punishment. 

We also aimed to establish which behaviours pet ferret owners suggest best indicate boredom compared to relaxed, fearful, or ‘happy’ ferrets. We hypothesised that, if boredom-like behaviours reflect an aversive sub-optimal arousal state [1], then pet ferret owners will suggest behaviours that incorporate both arousal-seeking behaviour (such as restlessness, distractibility, aggression, or escape behaviour) and low-arousal behaviours (such as yawning or lying awake) as boredom indicators in ferrets. Furthermore, if pet owner beliefs in boredom reflect their awareness of the affective state in ferrets, then pet owners who believe that ferrets experience boredom will have better agreement with the literature over which behaviours indicate a boredom-like state in ferrets than pet owners who express greater doubt as to whether ferrets can be bored. 

Finally, in this study, we aimed to describe which ferret management practices pet owners believe are most important for preventing boredom in their ferrets. Because boredom includes motivation for new sources of behavioural engagement or stimulation [3,34], we expected owners to suggest stimulating activities, such as novel exploration, sensory or interactive enrichment, and positive reinforcement training opportunities, rather than more restful environmental enrichment types.

## 2. Materials and Methods

An online survey was conducted from 13 February to 21 March 2020 using the hosting platform SurveyGizmo^TM^ (now known as Alchemer^®^). The survey is available as supplementary material within [13]. The survey was open to any English-speaking participants and was circulated to reach ferret caretakers from all sectors of ferret use. Only respondent data from the pet owner sector are discussed here (data from the other ferret caretaker sectors are discussed elsewhere: [13]). Pet owners were reached through posting links to the survey in Facebook^TM^ groups relating to ferret owners and through posting on the LinkedIn accounts of some of the authors. The survey was pilot tested by four ferret caretakers before release, and it received ethical approval from the Royal Veterinary College (URN SR2019-0441).

### 2.1. Survey Structure and Questions

The survey contained an introductory page informing participants of the broad survey aim to gather information on participants’ perceptions of ferret’s needs and preferences. Participants were informed that the survey data would contribute to ferret welfare research into how ferrets are kept and how they respond to their environment. No specific mention was made about ferret boredom or our hypotheses to help minimise bias from us leading them to answer in particular ways. Participants were also informed that the survey was anonymous, that they must be 18 years old or over to participate, that the survey completion took approximately 12 min, and that, in participating, they consented for the data to be used in this research. 

The survey comprised 36 questions split into three sections. The first section asked questions pertaining to demographics, the second to housing and enrichment provision (that section is described in greater detail in [13]), and the third to perceptions of ferrets’ affective states and behavioural indicators. Only one question, respondent age, was compulsory to help ensure all respondents whose data we retained for analyses were 18 years or over. A mixture of multiple-choice, short answer, and long answer formats were used, and ‘unsure’ was provided as an answer option wherever appropriate to avoid forcing indefinite answers.

#### 2.1.1. Section One: Respondent Demographics and Ferrets

Questions included respondent age, gender, years’ experience with ferrets, main ferret caretaker role (pet owner, working animals, laboratory, zoo, rescue, other), country of residence (if UK, respondents were asked to specify England, Northern Ireland, Scotland, or Wales), and the number and sex of ferrets currently in their care.

#### 2.1.2. Section Two: Ferret Housing, Environmental Enrichment, and Training

Questions included whether ferrets were housed individually or socially, the location of their housing (inside or outside), housing type and complexity (e.g., cage, hutch, or free-ranging; single or multi-level housing), and how many times per week ferrets were let out of their housing, and if so, for how long. Questions also asked what types of environmental enrichment were provided both inside and outside of the housing by selecting all that applied from a list; the enrichment options were randomly ordered for each respondent by the SurveyGizmo^TM^ software to minimise bias from order effects. Respondents were also asked how often they changed the enrichment offered to their ferrets. 

Respondents were asked whether they train their ferrets and for what purpose (e.g., basic handling, husbandry, tricks). Respondents were provided with 11 Likert item training scenarios and asked how likely they would act in the way the scenario outlined from ‘Extremely likely’ through to ‘Extremely unlikely’; the answer option ‘Not applicable’ was also provided. These training scenarios highlighted training techniques incorporating positive reinforcement (e.g., “You call your ferret to come to you and you reward her/him when they do”), negative reinforcement (e.g., “You are holding your ferret tightly because they are wriggling, but you relax your grip once she/he stays still”), negative punishment (e.g., “You are playing with a toy with your ferret. Your ferret starts to bite you during the play, so you remove the toy”), and positive punishment (e.g., “Your ferret bites you so you tap her/him on the nose”).

#### 2.1.3. Section Three: Respondent Perceptions of Ferret Affective States

Four questions were included, each asking respondents to choose what behaviours from a provided list they would expect to see from (1) a happy, (2) a relaxed, (3) a fearful or distressed, or (4) a bored ferret (Table 1). These states were selected as contrasting emotions to represent the four valence-arousal spaces [35], assuming that boredom is negatively valenced and of low arousal (albeit seeking higher arousal). 

Of the behaviours suggested, 13 were suggested in the literature [1,6,7,8] as potential behavioural indicators of boredom. These behaviours are indicated in Table 1 and are referred to as our ‘gold standard’ behavioural indicators of boredom. Respondents were kept blind as to which behaviours were the gold standard behaviours, which were potential distractors, and which might help distinguish boredom from the other states. The order in which the affective states and the behaviours were presented was randomized for each respondent by the SurveyGizmo^TM^ software.

To avoid signalling our specific interest in boredom and potentially leading respondents to answer in a biased way, questions pertaining to respondents’ beliefs in ferret boredom and ferret-boredom prevention were asked last in the survey. Questions then asked whether respondents think ferrets can experience boredom (a Likert item question ranging from ‘definitely’ to ‘definitely not’) with a free-text follow-up question asking the respondents to explain why they think this. Respondents were also asked whether they think the ferrets in their care had ever experienced boredom (yes/no) with a free-text follow-up question again asking respondents to explain why they think this. 

Finally, respondents were asked to rank a list of 11 answers to the question ‘what do you think is necessary to prevent boredom’ from most important to least important in preventing ferret boredom. The order of the answer options was randomised for each respondent by the SurveyGizmo^TM^ software to minimise order bias. The answer options were:

- Being outside;
- Having time to explore outside its home cage;
- Having tunnels or nesting areas;
- Interaction with familiar humans;
- Interaction with other species (e.g., other pets or animals);
- Nothing;
- Offering food in a bowl;
- Offering food so the ferret must work to access it;
- Social housing with other ferrets;
- Toys in their home cage, e.g., balls;
- Other (please specify).

### 2.2. Data Cleaning

Only respondents who selected ‘pet owner’ as their primary caretaker role were selected for analysis, because they comprised 82.4% of the responses [13]. Respondents who selected that they were under 18, or that they currently did not have a pet ferret, were removed from the analysis. All surveys that were labelled ‘partially completed’ by the SurveyGizmo^TM^ software, indicating the respondents abandoned the survey part way through, were excluded from the analysis. 

All statistics were carried out in the R software environment [49]. For answers to the short-answer free-text question ‘how much time do they usually spend out of the hutch/cage/enclosure when let out?’, if a range was provided, the middle value was used, and all answers were re-allocated to time-period categories. For multiple-choice questions where respondents selected ‘other’ and elucidated in the ‘please specify’ box, answers were re-allocated to one of the provided answer categories where this was reasonable (e.g., an answer of ‘taken for walks’ would be re-allocated to the existing answer category ‘Exploration of new areas’). 

The distribution of Likert item answers to the question ‘Do you think ferrets can experience boredom?’ was strongly skewed, so—to avoid excessively rare answers—they were grouped into two categories, with answers ‘definitely’ and ‘very probably’ grouped into the category ‘yes’ and answers ‘probably’, ‘possibly’, ‘probably not’, and ‘definitely not’ grouped into the category ‘doubt’. For the question ‘what do you think is necessary to prevent boredom’, to allow for different numbers of answers being included in the rank of importance by different respondents, we divided the total sum rank of each answer by the number of respondents who had selected it, providing an average rank position for each answer option. Multiple Likert item questions were asked pertaining to each of the different training styles. The Likert item answers were summed for each training style to create a Likert scale [50].

### 2.3. Data Analysis

The answers to two questions, Q1 (Likert scale): ‘do you think ferrets can experience boredom?’ and Q2 (yes/no): ‘do you think the ferrets in your care have ever experienced boredom?’, were used to indicate respondents’ perceptions of ferret boredom, and the analysis was carried out separately for each question. Firstly, a generalised linear model (GLM) with binomial distribution to test for relationships between respondents’ perception of ferret boredom and demographic variables (age/gender/ferret experience/location) was conducted. Backward stepwise selection of the full model was undertaken until the lowest Akaike Information Criterion (AIC), indicating the best model fit, was reached. Next, GLMs were run to test for relationships between respondents’ perceptions of ferret boredom and the ‘length of out-of-housing time ferrets received’ and the ‘number of environmental enrichment types provided’, which were the model dependent variables. Respondent opinion of ferret boredom (Q1 or Q2) was an independent variable alongside demographic variables in the full model. If significance was found in the full model, then backward stepwise selection of the model was undertaken until either all demographic variables were removed or the lowest AIC, indicating the best model fit, was reached. To test for relationships between respondents’ perceptions of ferret boredom and the categorical response variables ‘ferret housing type’ and the ‘frequency environmental enrichment was changed’, Fisher’s exact tests were conducted. Finally, Mann–Whitney U tests were conducted to test for relationships between respondents’ perceptions of ferret boredom and ferret training style (‘positive reinforcement’ and ‘positive punishment’ training). Statistical significance was set at *p* = 0.05, and the threshold for a statistical trend was set at *p* ≤ 0.10. To assess the standardized magnitudes of any significant effect sizes, we used Cohen’s d effect size calculations for the GLMs and Cliff’s delta effect size calculation for the Mann–Whitney tests.

To help describe the behaviours respondents associated with the four affective states (happy/relaxed/fearful/bored), a heat map of the behaviours was created using the ‘heatmap.2’ function in R package ‘gplots’ [51]. For boredom-behaviours, a Fleiss’ kappa statistic was run to check for the level of agreement between respondents (using the ‘kappam.fleiss’ function in the R package ‘irr’ [52]). Classification tree analysis using the ‘rpart’ function in the R package ‘rpart’ [53] was used to show the strength of probabilities of certain behaviours indicating the four affective states.

## 3. Results

### 3.1. Demographics

A total of 831 respondents completed the survey. Seventy-seven respondents were excluded, leaving 754 valid respondents, and of these, a total of 621 were pet owners. Pet owners were predominantly female (85.7%; Table 2) and predominantly from the UK (67.5%). Respondent age and experience with ferrets was widely distributed, with the age bracket 26–35 (28.3%) and the experience category of 1–5 years (37.7%) being the most commonly selected. Owning 3–6 ferrets (38.8%), followed by 2 ferrets (28.2%), were the most common number of pet ferrets (Table 2).

### 3.2. Pet Owner Perception of Ferret Boredom

A total of 582 respondents answered whether they believed ferrets in general can experience boredom, with the majority believing that ferrets are able to experience boredom (*n* = 541, 93.0%) and far fewer respondents expressing doubt as to whether ferrets can experience boredom (*n* = 41, 7.0%) (Table 3). Fewer respondents (*n* = 538) answered whether they believed their own pet ferrets had ever experienced boredom (yes/no), with 398 (74.0%) believing their ferrets had experienced boredom, and 140 (26.0%) believing their ferrets had never experienced boredom.

### 3.3. Associations between Pet Owners’ Perceptions of Ferret Boredom Versus Housing, Management, and Husbandry

**Figure 1:**
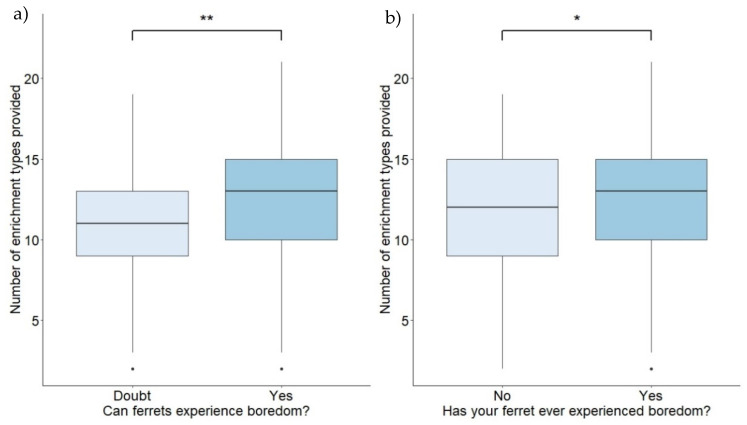
The number of environmental enrichment types provided by respondents who (**a**) expressed doubt as to whether ferrets can experience boredom, and those who believed ferrets can experience boredom, and (**b**) respondents who did not think their own pet ferrets had ever experienced boredom, and those who thought their pet ferrets had experienced boredom. * indicates a p value of less than 0.050, ** indicates a p value of less than 0.010.

Respondents who indicated doubt as to whether ferrets can experience boredom (Q1) provided significantly fewer types of enrichment than those who believed ferrets can experience boredom (GLM: t-value(1559) = 2.651; standard error = 0.604; *p* = 0.008) (median n environmental enrichment types for Yes = 13, and for Doubt = 11); a Cohen’s d calculation identified this as a small effect size (0.445, 95% confidence intervals (CI) [0.11, 0.78]) (Figure 1). Respondents who did not think their own ferrets had ever experienced boredom (Q2) also provided significantly fewer enrichment types than those who believed their ferrets had ever experienced it (GLM: t-value(1,517) = 2.518; SE = 0.363; *p* = 0.012) (Figure 1); Cohen’s d calculation again identified a small effect size (0.252, 95% CI [0.05, 0.45]).

**Figure 2:**
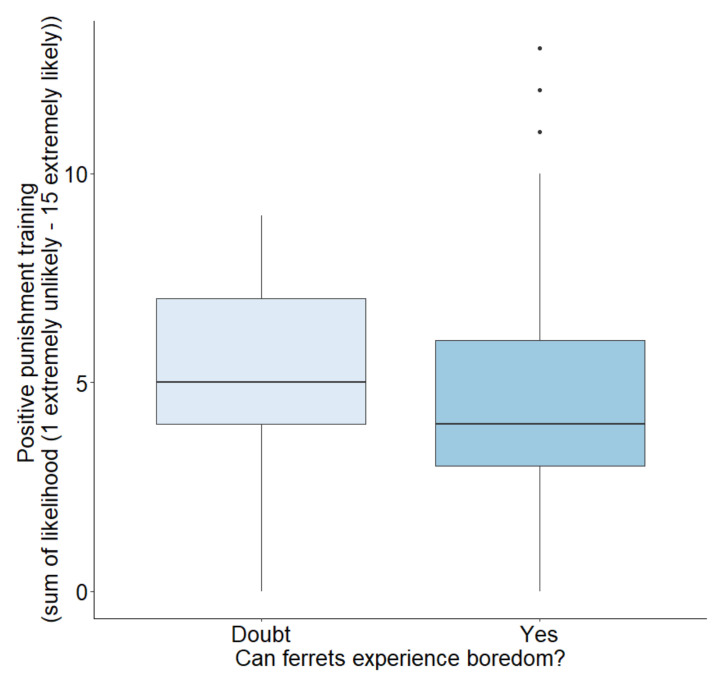
The likelihood of respondents who either doubted or believed that ferrets are able to experience boredom to use positive punishment-style training. The likelihood sum was taken from the answers to three Likert positive punishment training scenario questions, where respondents selected how likely they were to behave in the way detailed by the scenario, from ‘extremely likely’ through to ‘extremely unlikely’.

Training using positive punishment was not significantly associated with respondents’ beliefs that ferrets could experience boredom (Q1), but the trend was in the hypothesised direction, with respondents who doubted ferret boredom using slightly more positive punishment training (Mann–Whitney: W = 11,208, *p* = 0.081); a Cliff’s delta calculation identified this as a small effect size (0.219, 95% CI [0.05, 0.38]) (Figure 2).

No other associations between perception of ferret boredom (Q1 or Q2) and ferret training, housing type, or the amount of time ferrets were provided with out-of-house exploration opportunities were found. Boredom perception did not significantly predict the provision of any particular types of environmental enrichment, either. Respondent location was found to affect whether respondents believed that the ferrets in their care had ever experienced boredom (Q2) (GLM: Z(1472) = 3.431; S = 0.289; *p* = 0.001), and Tukey’s post hoc pairwise comparisons showed that respondents from the UK significantly more frequently answered that they believed their own ferrets had not experienced boredom than did respondents from both North America (Z = −3.431, SE = 0.289, *p* = 0.001) and the rest of the world (Z = −2.666, SE = 0.614, *p* = 0.019).

### 3.4. Behaviours That Pet Owners Associate with Ferret Boredom and Other Affective States

#### 3.4.1. Identification of Boredom

**Figure 3:**
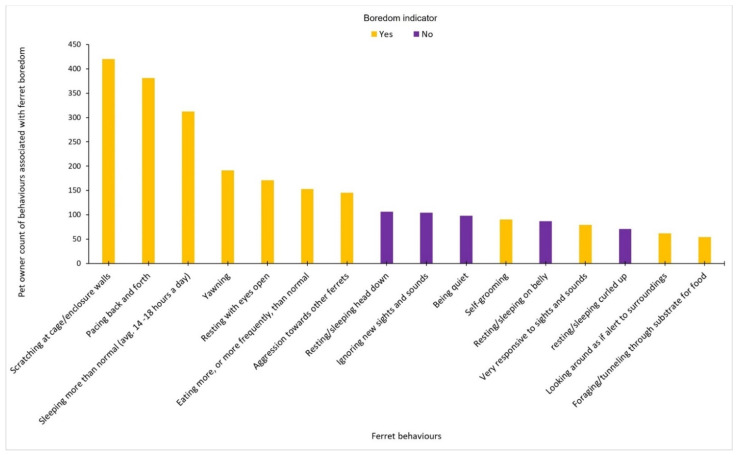
Behaviours that pet ferret owners associate with ferret boredom. Only behaviours selected by *n* > 50 respondents were included. Behaviours that are identified in the literature as being potential indicators of boredom are shown in gold, and those that are not potential indicators of boredom are shown in purple. Pet owners were not made aware of which behaviours were gold standard indicators of boredom.

The ten most selected behaviours that respondents associated with boredom in ferrets were scratching at cage or enclosure walls (73.81%), repetitive pacing (66.96%), sleeping more than normal (54.83%), yawning (33.57%), resting with eyes open (30.05%), eating more or more frequently than normal (26.89%), conspecific aggression (25.48%), resting or sleeping with head down (18.63%), ignoring new sights and sounds (18.28%), and being quiet (17.22%) (Figure 3). When the Fleiss’ kappa statistic was run to check the level of agreement between respondents, the level of agreement between respondents was ‘fair’ [54], K = 0.227, and greater than that would be expected by chance (Z = 581, *p* < 0.001). 

Of the 13 ‘gold standard’ behaviours (those identified from the literature as likely to be indicators of boredom; Table 1), the top seven behaviours most selected by respondents comprised these gold standard boredom indicators. However, only three gold standard indicators were selected by over 50% of the answering respondents (‘scratching at cage walls’, ‘pacing’, ‘sleeping more than average’) (Table 4). There was closer alignment between respondents and the gold standard for behaviours that were not suggested to indicate boredom, with 81.4–100.0% of respondents selecting behaviours as not being indicative of boredom in ferrets, which were also not suggested in the literature as being boredom indicators. Whether respondents believed or doubted whether ferrets could experience boredom was not found to affect whether they selected the gold standard behaviours identified as boredom indicators (Mann–Whitney: W = 8857, *p* = 0.111).

#### 3.4.2. Fear and Distress, Relaxation, Happiness, and Boredom

**Figure 4:**
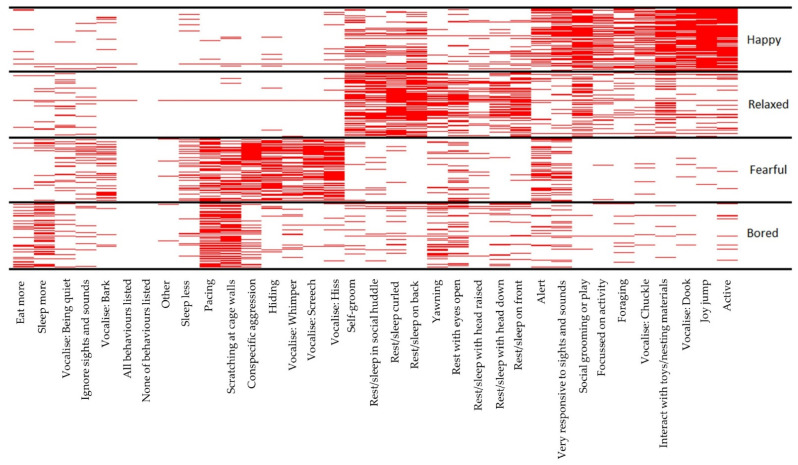
A heatmap showing the behaviours selected by respondents as occurring in ferrets in the four affective states: happy, relaxed, fearful, and bored. Where a respondent selected a behaviour as occurring in one of the states, a red line is shown. Where many respondents selected a behaviour as occurring in an affective state, the layering of red lines creates red bands. The greater the amount of red visible for a behaviour in a state, the more commonly that behaviour was selected as occurring in that state by respondents. The heatmap was clustered by the commonness of behaviour per affective state; thus, the behaviours most commonly selected by respondents in each state were grouped together to allow a clearer visualisation of the sets of behaviours associated with each affective state.

The numbers of respondents selecting behaviours as indicating each affective state were as follows: ‘happy’ *n* = 582, ‘fearful’ *n* = 580, ‘relaxed’ *n* = 576, ‘bored’ *n* = 569. Respondents selected the greatest number of behaviours for ‘happy’ and the least for ‘bored’: ‘happy’ (mean (interquartile range (IQR)) = 8.56 (6–11), ‘relaxed’ (7.25 (5–9)), ‘fearful’ (median (IQR) = 7 (4–9)), ‘bored’ (4 (3–7)). The behaviours that respondents selected for the four affective states showed a distinct pattern with respondents consistently selecting certain behaviours for each state (Figure 4). Behaviours commonly selected were ‘joy jumping’ and ‘being active’ for a happy ferret, ‘resting or sleeping curled up’ or ‘resting or sleeping on their back’ for a relaxed ferret, ‘hissing’ and ‘screeching’ vocalisations for a fearful ferret, and ‘scratching at cage walls’ and ‘sleeping more’ for a bored ferret. Some overlap of behaviours between states was observed, such as ‘social grooming or play’ being commonly selected for both happy and relaxed ferrets and ‘pacing’ or ‘scratching at cage walls’ often selected for both fearful and bored ferrets.

**Figure 5:**
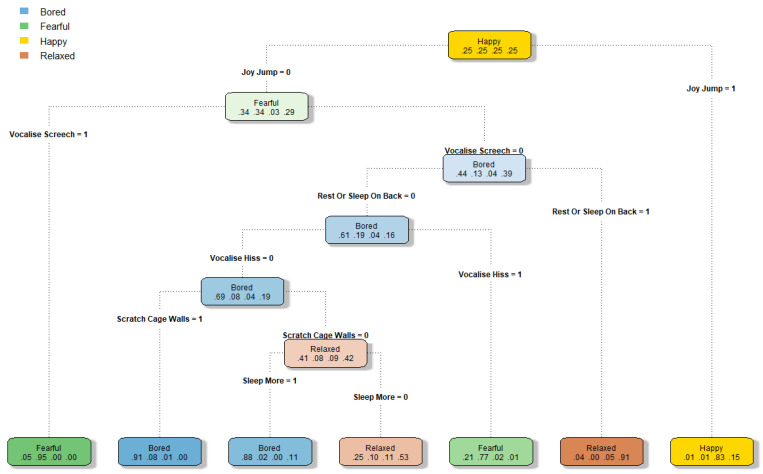
A classification tree showing the probability that respondents perceive a ferret to be experiencing either a bored, fearful, happy, or relaxed affective state. Coloured nodes indicate the most likely affective state at that point in the tree and show the probabilities of each of the states, with the numbers from left to right representing bored, fearful, happy, and relaxed. The branches represent behaviour, with 0 representing the absence of the behaviour and 1 representing the presence of the behaviour. For example, following from top to bottom, in the absence of ‘joy jump’ but the presence of ‘vocalise Screech’, there is a 5% probability that the ferret is perceived as bored, a 95% probability that the ferret is fearful, and a 0% probability that the ferret is happy or relaxed. The strength of the colour of each node reflects the certainty of the probability that the named affective state is the correct one. The percentages per node add up to approximately 100%, but this may not be exact due to rounding errors.

This distinctive patterning of behaviours with ferret affective state allowed the creation of a classification tree (Figure 5). The classification tree analysis identified the behaviours ‘joy jump’, ‘screech’, ‘resting or sleeping on their back’, ‘hiss’, ‘scratching at cage walls’, and ‘sleep more’ as the pivotal behaviours (based on the respondent data) in distinguishing which of the four affective states are most probable in a ferret. For example, following the classification tree in a top to bottom direction, if the ferret is not ‘joy jumping’, is not ‘screeching’, is not ‘resting or sleeping on their back’, is not ‘hissing’, but is ‘scratching on cage walls’, then owners are likely to classify the ferret as experiencing a boredom-like state with a 91% probability; and if that ferret is not ‘scratching at cage walls’ but is ‘sleeping more than usual’, then there is still an 88% probability that the owners would classify the ferret as bored.

### 3.5. Pet Owners’ Ranking of Ferret Boredom Preventers

Of the list of potential boredom preventers, respondents ranked housing with a conspecific as the most important for preventing boredom, followed by human tactile interaction, exploration of novel places or novel objects, spending time outside of their housing, and providing tunnels and nesting areas, in that order (Table 5). 

## 4. Discussion

The vast majority of ferret owners perceived their pets to have the capacity to experience boredom, and they mostly showed agreement with theoretical predictions about the behaviours suggested to be associated with ferret boredom. The owners who indicated doubt over whether ferrets can experience boredom provided significantly fewer environmental enrichment types, suggesting that ferret owners’ perceptions of their pets’ emotional capacity might directly affect the welfare of the animals in their care. The findings are discussed in more detail below.

### 4.1. Pet Owner Perceptions of Ferret Boredom

Most (93.0%) of pet ferret owners believed that ferrets are able to experience boredom and also perceived that their own pet ferrets had experienced boredom (74.0%). Similar results have been seen in other companion animal species. For example, in a survey of 194 participants of mixed animal and non-animal related backgrounds, most respondents believed pigs, dogs, cats, and cows to be capable of experiencing boredom, as well as hunger, pain, and fear [12]. Likewise, a survey of 687 horse owners and caretakers showed them to believe horses capable of experiencing boredom, as well as pain, fear, joy, and jealousy [11], although fewer horse owners and caretakers believed horses capable of experiencing boredom (65%) than ferret owners did in the current study. 

Human belief in animal boredom does not, of course, lend objective evidence that animals do genuinely experience boredom. Anthropomorphism could mean that people are interpreting animal behaviour as resembling that of bored humans in contexts that could reasonably be interpreted to be unstimulating or monotonous. This could mean nothing more than the animal behaviour superficially resembling human behaviour, without an equivalent emotion underlying it. However, deeper homologies do exist between human and animal behaviour, as evidenced, for example, by reliable and validated facial expressions in many mammals that indicate pain severity using equivalent facial muscles as those used in human pain expressions (reviewed in [55,56]). Therefore, it is possible that many pet owners recognise genuine homologies in the expression of boredom in their ferrets, and critical anthropomorphism could complement empirical evidence in understanding boredom-like states in animals [57,58].

Our findings may not be representative of ferret pet-owners in general due to the bias of female to male respondents (532:84, respectively) in this survey. Female respondents sometimes show greater awareness of, or belief in, an animal’s ability to have emotions than male respondents (e.g., [11,59]; although, for contrary findings, see [60]). Therefore, a more representative respondent pool may have resulted in a lower proportion of respondents believing ferrets capable of experiencing boredom. However, we did not find any significant gender effect on owners’ beliefs in the capacity for ferrets to experience boredom.

### 4.2. Associations between Pet Owners’ Perception of Ferret Boredom Versus Housing, Management, and Husbandry

Significantly fewer types of environmental enrichment were provided by respondents who expressed doubt over whether ferrets are able to experience boredom, suggesting that pet owners’ perceptions of ferret boredom relate to the welfare of the ferrets in their care, although the effect size was small. This finding is consistent with those of a rabbit survey, in which owners with a greater perception of rabbits ability to experience pain provided a greater number of enrichment types [17], which might suggest that people with a deeper understanding of their pets emotional capabilities may provide better welfare. It is possible that a reduced awareness of ferret affective capabilities may reflect a reduced awareness of a ferret’s needs more generally, which is reflected here in terms of environmental stimulation in the form of environmental enrichment. 

However, as this finding is correlational, we cannot be certain of any causal relationship between pet owners’ ferret boredom perception and environmental enrichment provision. Therefore, reverse causality is also possible, whereby pet owners who provide fewer types of environmental enrichment might have less opportunity to observe potential differences in their ferrets’ behaviour, which may occur with more diverse environmental enrichment types. Such behavioural differences may indicate a change in boredom-like state between lower and higher numbers of enrichment types, which are less apparent to pet owners, providing only low numbers of environmental enrichment types. 

Perhaps surprisingly, we did not find any relationship between boredom perception and the precise types of enrichment provided; thus, it is not necessarily the case that owners who perceive ferrets to experience boredom are any more likely to offer novel or stimulating enrichments to their ferrets compared with owners who doubt their ferrets’ boredom capacity. It was purely the number of types of enrichment that was associated. The provision of environmental enrichment is critical to the welfare of captive animals, providing stimulation and complexity to the environment, which can cause beneficial changes in behaviour (e.g., [61]), physiology (e.g., [62]), neurology (e.g., [63]), and affective state [6]. Higher numbers of enrichment items also lead to increases in ferret behaviours (dooking vocalisation, joy jumps or ‘weasel war dance’, and play) that are indicative of positive welfare states [26]. While there are likely to be multiple factors that influence a pet owners’ provision of environmental enrichment, our findings suggest that the perception of affective ability, specifically boredom, does positively correlate with owner provision of environmental enrichment and, consequently, pet ferret welfare. Owner attribution of emotional states does not always lead to improved welfare for the animal; for example, owners who attribute the capacity for guilt to their pet may punish the animal for ‘bad’ behaviour more than owners who do not [64]. Nevertheless, the correlation in the current study suggests that, if owners can be made aware of the potential for their pets to experience boredom, this could offer a means to encourage them to provide their animals with more environmental enrichment. 

While a significant relationship between ferret owners’ perceptions of ferret boredom and training style was not found, the trend was in the hypothesised direction, with owners who expressed doubt over ferret boredom seeming to use more punishment. If this trend is real, it is broadly consistent with a study of cat owners, in which owners who had less knowledge of cats used more punishment [19]. In a large, longitudinal survey of dog owners, male owners were more likely to use aversive training techniques than female owners [65]. Consequently, the relatively low number of male respondents in our survey could have diluted this finding in ferrets. However, this finding was only a trend; thus, it is not possible to rule out that this trend occurred by chance alone. Consequently, future research into the potential relationship between training style and ferret owners’ perception of ferret boredom with respondents with a more even gender ratio, and with a greater breadth of training style questions, is recommended.

### 4.3. Behaviours That Pet Owners Associate with Ferret Boredom and Other Affective States

The top five most commonly selected behaviours that owners associated with ferret boredom (scratching at cage or enclosure walls (73.8%), repetitive pacing (67.0%), sleeping more than normal (54.8%), yawning (33.6%), and resting with eyes open (30.1%)) are all behavioural indicators of boredom that were proposed from a theoretical basis (Burn, 2017). This suggests that many pet owners have an intuitive recognition of boredom in their ferrets that is consistent with these predictions, albeit with variation between respondents. Hötzel, Vieira, and Leme [11] similarly found that the behaviours horse owners and caretakers were associating with either pain, joy, or jealousy in horses aligned with the literature-supported behavioural indicators of those emotions. However, in the current study, while seven out of the thirteen identified gold standard behavioural indicators of boredom were in the respondents’ top 10 most frequently selected behaviours, only the top three behaviours (listed above) were selected by over 50% of respondents. Indeed, while there was agreement between respondents as to the behaviours they associated with boredom, the agreement was only ‘fair’ [54], and the alignment between respondents and the gold standard was closer for the behaviours not selected as associated with boredom (Table 4). 

Under 50% of respondents selected the low-arousal behaviours ‘yawning’ and ‘resting with eyes open’ as indicators of boredom; instead, these behaviours were more commonly selected as indicating relaxation. It is possible that neither yawning nor resting with eyes open are useful indicators of boredom in ferrets; thus, future experimental studies exploring the reliability of these behaviours as boredom-indicators are recommended. Alternatively, the behaviours may not have been as regularly selected as an indicator of boredom by respondents due to the stronger association of the behaviour with relaxation or other states not investigated here, such as tiredness [66]. Additionally, due to the fleeting nature of yawning, it may be rarely observed by owners. Similarly, the behaviour ‘resting with eyes open’ suggested to indicate boredom-like behaviour in ferrets [6] could be cryptic, especially to the casual observer, and it can possibly be mistaken for sleeping. For example, an owner may glance in the cage while walking by and, seeing their ferret lying down but not moving closer to confirm the ferret’s eyes are closed, think their ferret to be asleep. Therefore ‘resting with eyes open’ may have been captured in the commonly selected ‘sleeping more than normal’ behaviour. While there is evidence that ‘resting with eyes open’ indicates a boredom-like state in ferrets [6], there are mixed findings with the closely related American mink, with a meta-analysis concluding that it may be an unreliable boredom-indicator [5]; thus, further research into the suitability of this indicator in ferrets is recommended. 

The vocalisation ‘screeching’ has previously been linked to boredom in laboratory ferrets, with ferrets observed screeching more when in an unstimulating control condition compared to ferrets who received extra out-of-housing playtime [6]. However, pet owners rarely selected screeching as indicating ferret boredom (3.7% of respondents). Instead, screeching was identified during the classification tree analysis to be strongly classed as indicating a fearful or distressed ferret. Indeed, screeching vocalisations in ferrets are associated with pain, fear, or frustration [23,38,39]. This apparent inconsistency could be explained if the screeching observed by Burn, Raffle, and Bizley [6] was actually the result of aggression initiated by a bored ferret, but which caused screeching in the recipient (who may in that moment have been fearful rather than bored). Further, ferret boredom experiments are recommended to see whether the presence of screeching in a ferret housed in a way to induce boredom can be replicated to further identify whether this vocalisation is a useful boredom indicator.

Another surprising finding was that the behaviour ‘Ignoring new sights and sounds’ was the ninth most-selected behaviour by respondents as indicating boredom, and yet ‘Very responsive to sights and sounds’ and ‘Alert to surroundings’ were rarely selected as boredom indicators. Boredom leads to heightened sensation seeking behaviours and a desire for novelty, presumably to help break the monotony and escape the aversion of being bored (e.g., [6,27,67]); thus, we expected owners to identify an increased—not decreased—responsivity to stimuli as indicating boredom. It may be more likely that ‘Ignoring new sights and sounds’ represents apathy [8,48] rather than boredom. This highlights the difficulty in identifying animals’ affective states through cage-side behavioural observation alone.

The inconsistency in which respondents selected behaviours they would associate with boredom could reflect the challenging nature of measuring boredom. The first challenge is that boredom includes both low and high arousal behaviours [1] and the second is that many of the behaviours we may expect to occur in a bored animal may also occur in other affective states (e.g., escape behaviour with frustration [24]).This is apparent in the heatmap of behaviours that respondents associated with different affective states (Figure 4), with ‘pacing’ and ‘scratching at cage walls’ being commonly selected as occurring in both a bored and a fearful ferret. It is this diverse nature of boredom that makes it difficult to measure and observe. However, what is encouraging here is that the three behaviours that over 50% of respondents selected as occurring in a bored ferret comprised both low and high arousal behaviours (scratching at cage walls, pacing, and sleeping more than average). Similarly, the classification tree (Figure 5) showed both a high-arousal (scratching at cage walls) and a low-arousal (sleeping more) pathway to identify boredom in a ferret. 

Classification trees (or decision trees) use supervised machine learning algorithms to explore which features of the data, in this case ferret behaviours, are crucial to leading to each outcome, or in this case affective state [68]. Our classification tree is an example of how survey data could be used to help inform or corroborate animal affective state research through the identification of behavioural indicators. For example, if we assume that owner interpretations of their ferrets’ emotional states are mostly valid, our results suggest that the absence of joy jumping and the presence of vocal screeching are likely to reflect fearfulness with high probability; on the other hand, the presence of scratching at cage walls or sleeping more, in the absence of joy jumping, screeching, resting or sleeping on their back, and hissing, are likely to reflect boredom with high probability. Such a tool could be used both by welfare scientists and ferret caretakers, in future studies, in a way similar to the decision trees used in clinical settings for identifying an illness from the absence or presence of certain symptoms (e.g., [69]).

### 4.4. Pet Owners’ Ranking of Ferret Boredom Preventers

Pet ferret owners ranked housing with a conspecific, human tactile interaction, exploration of novel places or novel objects, spending time outside of their housing, and providing tunnels and nesting areas as most important for preventing boredom, in that order. Many of these management practices and environment enrichment types were those that were already commonly provided by these respondents, with human tactile interaction, tunnels, and the exploration of new areas being the in the top five most commonly provided enrichments (with 74.2%, 67.8%, and 67.8% of pet owners providing these, respectively) (analysed elsewhere, [13]). Whilst it is possible that owners were already providing those stimuli with the aim of helping prevent boredom, it is also possible that owners were simply suggesting their existing provision because those were what they had the experience of providing, and the suggestion of them being used to mitigate boredom was a retrospective evaluation. It is possible that certain less commonly provided experiences could also help mitigate boredom. For example, owners reported that their ferrets seemed to greatly enjoy digging opportunities and scent trails, which were rarely provided to ferrets [13].

Unexpectedly, tunnels and nesting materials were ranked above other stimulating enrichments, including training opportunities, exploring outside, working for food, and a varied diet. The high ranking of tunnels and nesting materials as a boredom preventer is surprising, as they lack the novelty and stimulation provided by the other highly ranked management practices. However, ferrets are a burrowing species; thus, it is possible that the provision of many tunnels could provide a behaviourally relevant, complex, and stimulating environment for ferrets. Ideally, tunnels and nesting materials should have been separate answer options in this questionnaire, because, whilst tunnels are not necessarily used by ferrets as resting places, nesting materials are instead important for resting and sleeping in. Whilst boredom can include low-arousal behaviours, by definition, boredom would not be expected to be relieved by resting, but instead by the opportunity to engage in more stimulating activities.

Research into what management practices can truly mitigate boredom is in its infancy, but our results offer some suggestions from a sample of pet owners as to which practices may show the most promise. Social housing, human tactile interaction, the exploration of new places or objects, and time outside of housing are all management elements that are likely to provide stimulation and variety for ferrets and would likely be effective mitigation against boredom. It is important to note that this is not an exhaustive list of boredom preventors, as we only provided respondents with a list of 13 potential boredom-mitigation strategies to rank. In fact, there are likely to be many more strategies that could be useful boredom preventors, such as offering digging opportunities and scent trails or housing in a complex environment [13]. We recommend future research to test the effectiveness of these boredom preventers in reducing signs of boredom in ferrets.

## 5. Conclusions

The majority of pet ferret owners believed their ferrets to be capable of experiencing boredom. Where pet owners expressed doubt over their ferret’s ability to experience boredom, they provided significantly fewer environmental enrichment types, likely negatively impacting their pet ferret’s welfare. This suggests that raising awareness of ferrets’ emotional capabilities with pet owners could be a viable approach to improving ferret management and welfare. 

Additionally, pet ferret owners were able to identify many of the behaviours predicted to indicate ferret boredom, as well as relaxation, happiness, and fear or distress. Yet, there was a discrepancy between owner suggestions and suggestions from the literature for certain indicators, specifically, ‘yawning’, ‘resting with eyes open’, ‘responsivity to the environment’, and ‘screeching’. Further investigation into the validity of these behavioural indicators of ferret boredom in experimental settings is recommended. 

This study also provides a useful list of boredom preventers that could be utilised by pet ferret owners looking for ways to prevent or reduce boredom in their pet ferrets. The efficacy of these boredom preventers at mitigating boredom in ferrets should be further explored in experimental settings.

## Figures and Tables

**Table 1:** The list of behaviours respondents could select from for the questions ‘Which behaviours might you expect to see from a [happy/relaxed/fearful or distressed/bored] ferret?’. The questions were presented in a tick-all-that-apply format. Behaviours identified from the literature as being potential boredom-indicators (gold standard boredom indicators) are indicated (yes/no); this information was not shown to respondents. The order these behaviours were presented in was randomised for each respondent. Where potential behavioural indicators of boredom may also indicate alternative affective states, these are listed. Additionally, for behaviours that are not considered indicators of boredom, but which may reflect other affective states, these states are indicated.

Behaviour Category	Gold Standard Boredom Indicator	Behaviour	Supporting Reference/s as a Boredom Indicator	Alternative Affective States Behaviour May Indicate	Supporting Reference/s as an Indicator of an Alternative State
Resting postures	Yes	Resting/sleeping head raised	[6,7,8,36]	Relaxation	[37]
	No	Resting/sleeping on back with belly exposed	-	-	-
	No	Resting/sleeping on belly	-	-	-
	No	Resting/sleeping curled up	-	-	-
	No	Resting/sleeping head down	-	-	-
	No	Resting/sleeping huddled with cage mate.	-	-	-
Vocalisations	Yes	Scream/screeching	[6]	Fear/anger/frustration/pain	[38,39]
	No	Dook	-	Excitement/joy	[38,39]
	No	Chuckling	-	Excitement/joy	[38,39]
	No	Barking	-	Fear/excitement	[39]
	No	Hissing	-	Anger (warning)/fear	[38,39]
	No	Whimpering/whining	-	Pain	[38]
	No	Being quiet	-	-	-
Behaviours	Yes	Aggression towards other ferrets	[1]	Anger/fear	[40,41]
	Yes	Eating more, or more frequently, than normal	[1,8]	-	-
	Yes	Foraging/tunnelling through substrate for food	[1,8]	-	-
	Yes	Looking around as if alert to surroundings	[1]	Anxiety (vigilance)	[42]
	Yes	Pacing back and forth	[1,43]	Frustration	[24]
	Yes	Resting with eyes open	[6,7,8,36]	-	-
	Yes	Scratching at cage/enclosure walls	[1]	-	-
	Yes	Self-grooming	[1,44]	Stress/anxiety	[45]
	Yes	Sleeping more than normal (avg. 14–18 h a day) *	[1]	-	-
	Yes	Very responsive to sights and sounds	[1]	-	-
	Yes	Yawning	[1]	Fear/stress/anxiety	[46,47]
	No	Active behaviours such as running, digging, exploring	-	-	-
	No	Dance of joy	-	Excitement/joy	[38]
	No	Ferret war dance	-	Excitement/joy	[38]
	No	Focused on what it is doing	-	-	-
	No	Grooming or playing with other ferrets	-	-	-
	No	Hiding	-	-	-
	No	Ignoring new sights and sounds	-	Apathy	[48]
	No	Interacting with enrichment (e.g., toys or nesting materials)	-	-	-
	No	Sleeping less than normal *	-	-	-
	No	All of the above	-	-	-
	No	None of the above	-	-	-
	No	Other (please specify)	-	-	-

* Time asleep may be reduced by boredom [1]. For the purposes of this survey, the behaviour ’sleeping more than normal’ was suggested as a boredom indicator to encapsulate more subtle low-arousal behaviour, e.g., lying awake but inactive, in combination with sleeping, where the distinction may be less apparent to the casual observer.

**Table 2:** Pet ferret owner (survey respondents) demographics, including gender, location, age, experience with ferrets, and number of pet ferrets.

Demographic	Category	Count
Gender	Female	532
	Male	84
	Other gender	5
Location	UK	419
	North America	149
	Rest of the world	48
	*Unanswered*	5
Age	18−25	120
	26−35	176
	36−45	154
	46−55	111
	56−65	52
	Over 65	8
Experience with ferrets	<1 month	8
	1−12 months	84
	1−5 years	234
	6−10 years	129
	>10 years	163
	*Unanswered*	3
Number of pet ferrets	1	86
	2	175
	3−6	241
	7−10	53
	11−50	51
	51−100	4
	*Unanswered*	11

**Table 3:** Pet owner responses to the question ‘Do you think ferrets can experience boredom?’. During data cleaning, the Likert answer options were grouped into the categories ‘Yes’ and ‘Doubt’.

Grouped Categories	Likert Answer Options	Count
Yes	Definitely	483
	Very probably	58
Doubt	Probably	20
	Possibly	14
	Probably not	6
	Definitely not	1
*Unanswered*	*Unanswered*	39

**Table 4:** The behaviours offered to respondents to select from as occurring in a bored ferret or not. Results are listed in order by % of respondents agreeing with the gold standard (Yes/No) status of the behaviour as an indicator of boredom. For gold standard behaviours that indicate boredom (Yes), the % represents respondents who selected the behaviour as being a boredom indicator. For behaviours that are not gold standard boredom indicators (No), the % represents respondents who did not select the behaviour as being an indicator of boredom. Behaviours are shown in bold font where over 50% of the respondents agreed with the gold standard. The total number of respondents answering this question was 569.

Gold Standard Indicator of Boredom	Behaviour	Agree with Gold Standard (%)	Agree with Gold Standard (*n*)
Yes	Scratching at cage walls	73.8	420
	Pacing	67.0	381
	Sleeping more than average	54.8	312
	Yawning	33.6	191
	Resting with eyes open	30.1	171
	Eating more	26.9	153
	Conspecific aggression	25.5	145
	Self-grooming	15.8	90
	Very responsive to sights and sounds	14.1	80
	Alert to surroundings	11.1	63
	Foraging	9.5	54
	Resting/sleeping with head raised	8.3	47
	Screech vocalisation	3.7	21
No	All the behaviours listed	100.0	569
	None of the behaviours listed	99.3	565
	Dook vocalisation	98.9	563
	Joy jumping	98.6	561
	Focused on activity	98.4	560
	Chuckle vocalisation	98.2	559
	Bark vocalisation	96.8	551
	Resting/sleeping on back	96.5	549
	Social grooming or play	95.8	545
	Sleeping less than average	95.6	544
	Interacting with toys or nesting materials	94.7	539
	Active behaviours	94.0	535
	Hiss vocalisation	92.8	528
	Whimper vocalisation	92.1	524
	Hiding	91.7	522
	Resting/sleeping in a social huddle	91.7	522
	Resting/sleeping curled up	87.5	498
	Resting/sleeping on front	84.7	482
	Being quiet	82.8	471
	Ignoring sights and sounds	81.7	465
	Resting/sleeping with head down	81.4	463

**Table 5:** Potential ferret boredom preventers as ranked by respondents to be most important for preventing ferret boredom (a rank of 1 for the most important to 13 for the least important). Not all boredom preventer answers were included in the ranking by all respondents. The boredom preventers are listed in order from the highest ranked (most important for preventing boredom) to the lowest ranked (least important for preventing boredom).

Boredom Preventer	Number of People Selecting This Answer (Total *n* = 527)	The Sum of the Scores (1−13) Attributed to This Answer	Ranked Boredom Preventers
Conspecific housing	504	898	1.8
Human tactile interaction	515	1472	2.9
Exploration of novel places or objects	503	1989	4.0
Time outside of housing	488	2016	4.1
Tunnels and nesting areas	498	2881	5.8
Toys inside home cage	474	3122	6.6
Training and learning	376	2591	6.9
Being outside	380	2724	7.2
Working to access food	345	2657	7.7
Interaction with other species	294	2320	7.9
Varied diet	359	2893	8.1
Food in a bowl	276	2689	9.7
Nothing	103	1332	12.9

## Data Availability

The data presented in this study are available upon request from the corresponding author. The data are not publicly available due to participant consent to their data being used for research only of the nature described in the questionnaire introduction.

## Supplementary Materials

The following supporting information can be downloaded at: https://www.mdpi.com/article/10.3390/ani12233262/s1, https://www.mdpi.com/2076-2615/12/9/1065#supplementary, Questionnaire S1: Ferrets: A survey of housing, management, and welfare.
